# In-sewer iron dosing enhances bioenergy recovery in downstream sewage sludge anaerobic digestion: The impact of iron salt types and thermal hydrolysis pretreatment

**DOI:** 10.1016/j.wroa.2024.100273

**Published:** 2024-10-29

**Authors:** Jingya Xu, Yizhen Wang, Yanzhao Wang, Lai Peng, Yifeng Xu, Hailong Yin, Bin Dong, Xiaohu Dai, Jing Sun

**Affiliations:** a State Key Laboratory of Pollution Control and Resource Reuse, School of Environmental Science and Engineering, Tongji University, Shanghai, 200092, PR China; b Key Laboratory of Yangtze River Water Environment, School of Environmental Science and Engineering, Tongji University, Shanghai, 200092, PR China; c Shanghai Institute of Pollution Control and Ecological Security, Shanghai, 200092, PR China; d Key Laboratory of Green Utilization of Critical Non-metallic Mineral Resources, Ministry of Education, Wuhan University of Technology, Wuhan 430070, PR China

**Keywords:** Iron salts dosing, Sewer system, Bioenergy recovery, Anaerobic digestion, Methane, Ferrate

## Abstract

Dosing iron salts is a widely adopted strategy for sewer odor and corrosion management, and it can affect bioenergy recovery during anaerobic digestion (AD) of sludge in downstream wastewater treatment plants. However, the different impacts of in-sewer iron salt dosing on AD, depending on the types of iron and digestion conditions, remain unclear. Therefore, this study investigated the impact of in-sewer ferrous (Fe(II)) and ferrate (Fe(VI)) dosing on bioenergy recovery in both conventional AD and AD with thermal hydrolysis pretreatment (THP). The results showed that in-sewer Fe(VI) dosing notably enhanced methane production in AD more than in-sewer Fe(II) dosing, with cumulative methane yields of 197.1±1.9 mLCH_4_∙gVSadded^−1^ for Fe(VI) and 186.5±10.4 mLCH_4_∙gVSadded^−1^ for Fe(II), respectively. Microbial analyses and iron particle characterizations suggested that the superior promotion with Fe(VI) dosing may be attributed to the smaller particle sizes and higher iron oxide content of Fe(VI) resultant products. This led to a greater enhancement in direct interspecies electron transfer (DIET) between syntrophic bacteria and methanogens, as indicated by the upregulation of *Methanosaeta* and key functional genes involved in CO_2_-utilizing methanogenesis. Additionally, in THP-AD, the methane production enhancement caused by in-sewer iron dosing (35.5 mLCH_4_∙gVSadded^−1^) exceeded that in conventional AD (26.9 mLCH_4_∙gVSadded^−1^), although organic degradation during THP was unaffected. As THP-AD gains popularity for improved bioenergy recovery from sludge, our findings suggest that in-sewer iron dosing supports this advancement. Furthermore, in-sewer Fe(VI) dosing appears more promising within integrated wastewater management strategies, facilitating energy- and carbon-neutralization of urban water systems.

## Highlights

• Both in-sewer Fe(II) and Fe(VI) dosing can improve bioenergy recovery from sludge.
• In-sewer Fe dosing mainly enhanced DIET during sewage sludge anaerobic digestion.
• Small size and high iron oxide content of Fe(VI) resultant particle favored the improvement.
• The improvement caused by in-sewer Fe dosing was further enhanced in THP-AD.
• In-sewer Fe dosing is conducive to energy neutralization of urban water systems.

## Graphical abstract

**Figure:**
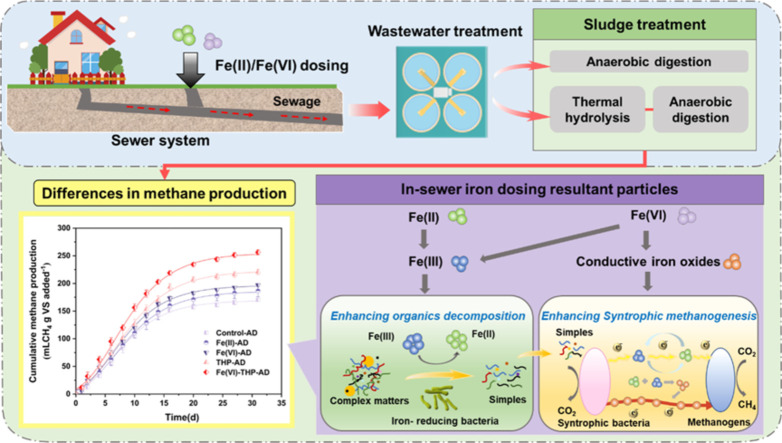

## 1 Introduction

Sewers are important urban infrastructures providing essential protection for public health (Pikaar et al., 2014). However, sewer assets always face serious threats during long-term operations. Sulfide-induced corrosion can lead to structural collapses and consequently heavy financial burdens for rehabilitation or replacement (Jiang et al., 2015; Pikaar et al., 2014). In addition, hydrogen sulfide emitted from sewers always cause odor complaints in neighborhoods and even poses life-threatening risks to sewer workers due to its toxicity (Jiang et al., 2015, Sun et al., 2014a, Sun et al., 2014b, Wang et al., 2013). A current method to cope with sulfide-induced problems is adding different chemicals to sewers. Oxidizers (oxygen/air/nitrate) (Ganigué et al., 2014; Gutierrez et al., 2008; Jiang et al., 2013a), iron salts (ferric chloride/ferrous chloride) (Kulandaivelu et al., 2020; Rebosura et al., 2018), and biocides (free nitrous acid/hydrogen peroxide) (El Brahmi et al., 2023; Jiang et al., 2011), have been applied to either suppress sulfide production or remove sulfide once generated. However, once added into sewers, these chemicals or resultant products can reach downstream wastewater treatment plants (WWTPs) with flow and may affect the subsequential treatment processes.

Iron salts are among the most frequently used chemicals for in-sewer sulfide control. In Australia, the volume of wastewater receiving iron salts (Fe (II) /Fe (III)) was reported to account for ∼66% of the total wastewater subjected to in-sewer chemical dosing (Ganigue et al., 2011). A recent study showed that the iron-containing solid (mainly FeS) generated after in-sewer FeCl_2_ dosing could enter the downstream WWTP and be beneficial to sludge anaerobic digestion (AD) (Kulandaivelu et al., 2020). More recently, ferrate (Fe(VI)), a hexavalent iron salt, has been proposed to control sulfide in sewers (Yan et al., 2020; Yan et al., 2023). Different from Fe(II) and Fe(III), which mainly react with sulfide already generated (Yang et al., 2024), Fe(VI) was found to have a rapid biocidal effect on microorganisms in sewer biofilms, thus preventing sulfide generation. However, the iron-containing solids generated after Fe(VI) addition can be different from those generated after Fe(II) or Fe(III) addition, as FeS would not be largely produced in the Fe(VI) addition case. The particle sizes and morphologies of the solids may also be varied. Such differences may pose distinct impacts on the downstream sludge AD. However, it remains unclear to date.

Meanwhile, various pretreatment approaches have been developed to improve bioenergy recovery from sludge AD (Tian et al., 2022; Wu et al., 2021a; Zou et al., 2018). Thermal hydrolysis pretreatment (THP) is one of the most commonly used approaches and has been applied in nearly 30 countries (Kor-Bicakci et al., 2019). The THP process destroys the sludge structure and microbial cells through heating and high pressure so that the methanogenic potential and biodegradability of the sludge can be improved (Barber, 2016). However, previous studies only investigated the effect of in-sewer iron addition on conventional AD. With the growing emphasis on energy- and carbon-neutralization in urban water management (Rabaia et al., 2024), pretreatment methods like THP are increasingly applied in WWTPs. Therefore, the impact of in-sewer iron addition on AD with THP should be investigated for a more comprehensive understanding.

Thus, this study aims to systematically evaluate the impact of in-sewer iron dosing on downstream sludge AD, specifically focusing on different iron salts (Fe(II) and Fe(VI)) that were used and whether THP pretreatment is applied. This is because Fe(II) and Fe(VI) followed distinctive mechanics in controlling sulfide production in sewers and THP is one of the most widely used pretreatment methods for sludge AD as stated above. The biochemical methane production under different conditions was compared, and organic matter conversion at different stages was tracked. Moreover, the microbial communities and functional genes in the digestor as well as the characteristics of the resultant iron-containing solid were investigated to explore the underlying mechanisms of different impacts. This study is expected to fill the knowledge gap of current understanding about the effects of in-sewer iron dosing on the downstream sludge AD so as to facilitate the development of iron-based integrated strategies for energy- and carbon-neutralization in urban water management.

## 2 Results

### 2.1 Methane production from sludge anaerobic digestion under different in-sewer iron dosing and pretreatment conditions

**Fig. 1:**
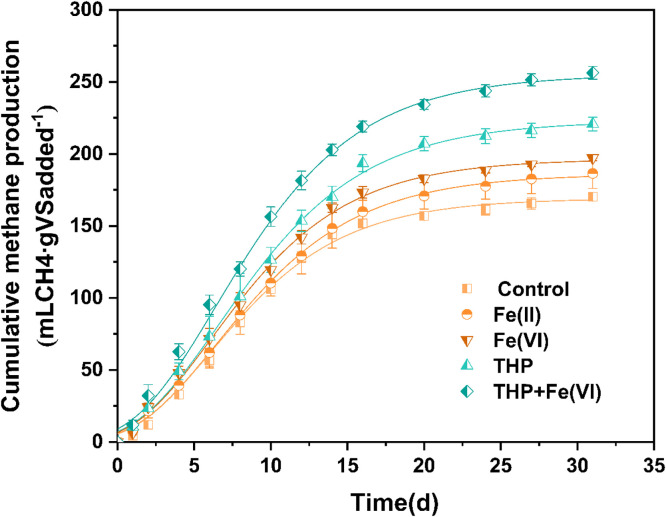
Cumulative methane production in anaerobic digestors operated under different in-sewer iron dosing and pretreatment conditions. The dots referred to the experimentally measured data and the lines indicated the simulated methane production using a modified Gompertz model.

The methane production from sludge AD under different upstream in-sewer iron dosing conditions was monitored over 30 days. The results (Fig. 1) showed that the in-sewer iron dosing effectively enhanced cumulative methane production (P<0.05), with the values being 186.5±10.4 mLCH_4_∙gVSadded^−1^ for Fe(II) dosing, 197.1±1.9 mLCH_4_∙gVSadded^−1^ for Fe(VI) dosing, and 170.2±2.6 mLCH_4_∙gVSadded^−1^ for the control group. Furthermore, the in−sewer Fe(VI) dosing exhibited a greater promotion effect than Fe(II) dosing (P<0.05), as the methane production was enhanced by 1.15 and 1.05 times for Fe(VI) dosing and Fe(II) dosing, respectively. The methane production was well described by the modified Gompertz model (Fig. 1, R^2^>0.99). The parameter estimation (Table 1) suggested that the in-sewer iron dosing could enhance the maximum methane production rate (R_max_) by 4.0%-13.5% and the methane production potential (G_0_) by 10.0%-16.2%. This indicated that in-sewer iron dosing favored both microbial growth and sludge degradation in an anaerobic digestor.

**Table 1:** Estimated G_0_, R_max_, and λ values (with 95% confidence intervals) in different anaerobic digestion reactors using the modified Gompertz model.

	Methane production potential (G_0_) (mL/(g VS·d))	Maximum methane production rate (R_max_) (mL/(g VS·d))	Lag phase(λ) (day)
Control	169.1±2.7	12.6±0.6	1.1±0.3
Fe(II)	186.0±1.9	13.1±0.4	1.3±0.2
Fe(VI)	196.5±2.6	14.3±0.5	1.1±0.2
THP	222.9±2.7	15.2±0.5	1.3±0.2
THP+Fe(VI)	255.1±3.7	17.7±3.7	1.1±0.3

The methane production from sludge AD with THP under in-sewer iron dosing conditions was also tested. As shown in Fig. 1, the THP increased cumulative methane production by 30%, reaching 220.8±4.8 mL CH_4_∙gVSadded^−1^, and the Fe(VI) dosing in upstream sewers further increased the cumulative methane production to 256.3±4.5 mLCH_4_∙gVSadded^−1^. The absolute increase in methane production caused by in-sewer iron dosing in THP-AD (35.5 mLCH_4_∙gVSadded^−1^) is higher than that achieved in conventional AD (26.9 mLCH_4_∙gVSadded^−1^). The modeling analysis (Table 1) further showed the relative improvement of R_max_ by in-sewer Fe(VI) dosing in THP-AD (16.4%) was higher than that achieved in conventional AD (13.5%), suggesting that with the THP treatment, the microbial activities were improved more significantly by in-sewer iron dosing.

### 2.2 Impact of in-sewer iron dosing on carbon transformations at different stages

**Fig. 2:**
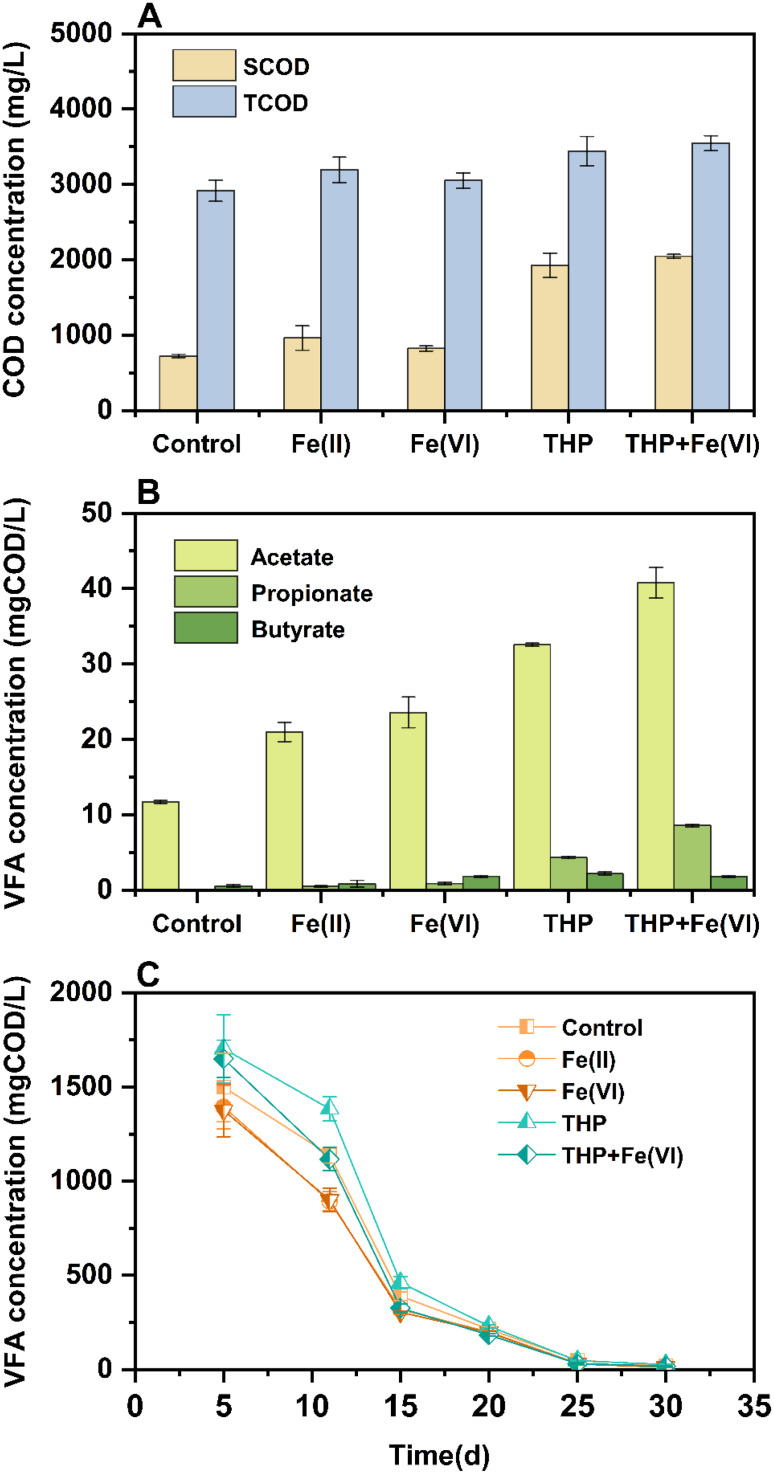
(A) TCOD and SCOD concentrations in the sludge before feeding the anaerobic digestor. (B) VFA concentrations in the sludge before feeding the anaerobic digestor. (C) Changes in VFA concentration during anaerobic digestion.

Differences in methane production improvement shown in Section 2.1 could be related to variations in carbon transformation in tested systems. Therefore, firstly, the volatile fatty acid (VFA), soluble chemical oxygen demand (SCOD), and total chemical oxygen demand (TCOD) concentrations were measured in the feeding sludge before AD to evaluate how in-sewer iron dosing affected carbon transformation during activated sludge processes and THP. As shown in Fig. 2A, the in-sewer iron dosing significantly increased VFA concentrations in the feeding sludge. Specifically, as a predominant VFA, acetate concentrations increased by 1.7 and 2.0 times with in-sewer Fe(II) and Fe(VI) dosing (P<0.01), respectively. The increase of propionate and butyrate concentrations can also be observed with in-sewer iron dosing (P<0.05), although their concentrations were below 2 mgCOD L^−1^ in all cases. The VFA in the feeding sludge was probably produced during the sedimentation and the storage period before feeding to the anaerobic digestor, as the anaerobic condition could occur. The iron in the sludge could enhance the organic decompositions through microbial iron reduction and therefore the VFA concentration was increased (Hao et al., 2017; Ye et al., 2022). In contrast, the SCOD and TCOD of the feeding sludge were not significantly affected (P>0.05, Fig. 2B). When the THP was applied, the VFA concentrations increased by 26.8 mg/L for sludge without in-sewer iron dosing. While receiving in-sewer Fe(VI) dosing, THP increased the VFA concentration by a comparable level of 24.9 mg/L. This similar enhancement suggested that in-sewer iron dosing did not significantly affect VFA transformations during the THP process (Choi et al., 2018).

The VFA profiles during sludge AD in different cases were also monitored (Fig. 2C) and the concentrations gradually decreased from Day 5 to Day 30 in all reactors. In conventional AD, the control reactors showed a higher VFA accumulation at Day 5 (1498.5±181.5 mgCOD/L) and a lower VFA decreasing rate especially during 5–10day (60.0 mgCOD∙L^−1^∙day^−1^) compared with the reactors receiving sludge with in-sewer iron dosing (1394.3±115.1–1376.2±140.0 mgCOD/L, 79.4∼83.6 mgCOD∙L^−1^∙day^−1^). A similar trend was also observed in THP-AD groups. The VFA consumption rate was increased in the in-sewer iron dosing reactor. Specifically, the VFA consumption rate was 89.0 mgCOD∙L^−1^∙day^−1^ in THP-AD with in-sewer Fe(VI) dosing and 53.1 mgCOD∙L^−1^∙day^−1^ in THP-AD without in-sewer iron dosing. These results were in good accordance with the improved methane productions caused by in-sewer iron dosing as shown in Section 2.1

### 2.3 Variation in microbial communities and metabolic pathways in anaerobic digestor under different in-sewer iron dosing and pretreatment conditions

#### 2.3.1 microbial communities

**Fig. 3:**
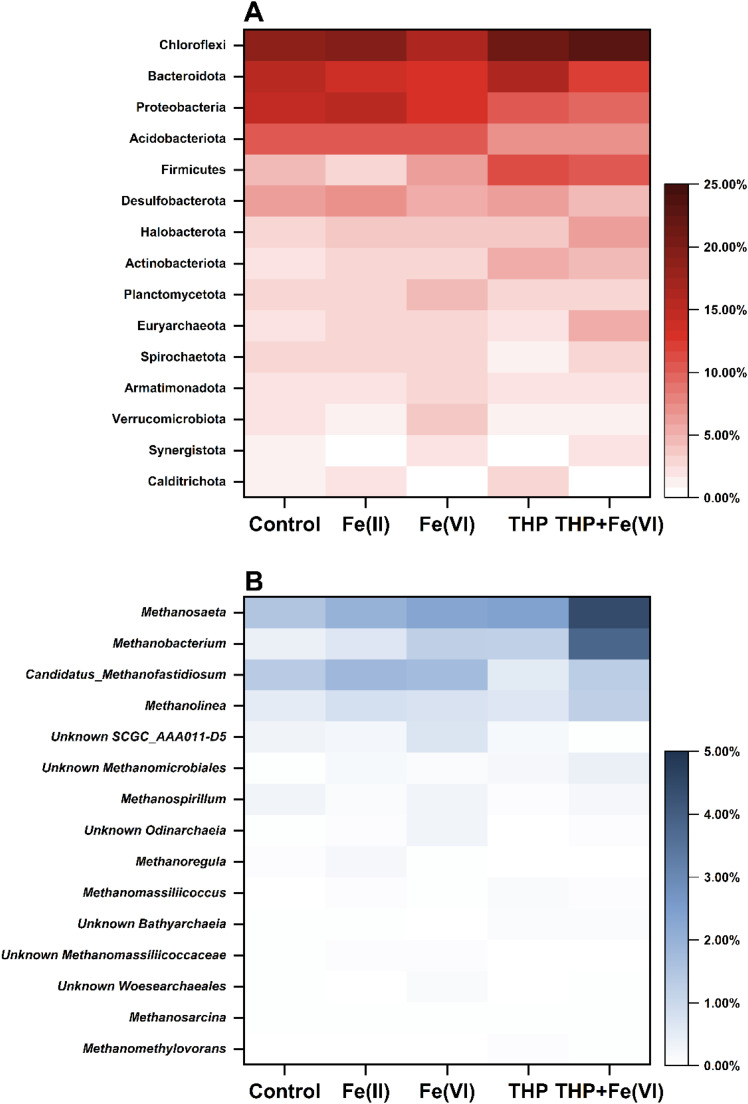
Microbial community structures in different anaerobi digestion reactors. (A) Heatmap showing the relative abundances of the top 15 most abundant microorganisms at phylum level; (B) Heatmap showing the relative abundances of the top 15 genera in Archaea domain.

The microbial community structures in anaerobic digestors under different in-sewer iron dosing and pretreatment conditions were analyzed using high-throughput 16S rRNA sequencing. The top 15 dominant phyla in digestors were compared in Fig. 3A. In general, the in-sewer iron dosing significantly enhanced the relative abundance of Halobacterota and Euryarchaeota, but decreased the relative abundances of Bacteroidota. For other phyla, in-sewer Fe(II) and Fe(VI) dosing posed different enrichment effects, as in-sewer Fe(II) dosing enriched Chloroflexi, Proteobacteria, and Spirochaetota, whereas Fe(VI) dosing enriched Firmicutes, Planctomycetota, Armatimonadota and Verrucomicrobiota. More specifically, the abundance of Halobacterota and Euryarchaeota, typical Archaeal phyla containing various genera of methanogens, increased by 1.35 and 1.46 times, respectively, for anaerobic digestors receiving in-sewer Fe(II) dosing, and a further improvement of 0.1 and 0.27 times were achieved in Fe(VI) case. For THP-AD, the relative abundance of Halobacterota and Euryarchaeota were enhanced more significantly by in-sewer Fe(VI) dosing, with increases of 1.86 and 2.90 times, respectively. Such enrichment was well aligned with the improvement of microbial activities as presented in Section 2.1.

The variations of Archaea at the genus level were further compared in Fig. 3B. *Methanosaeta, Methanobacterium, Candidatus_Methanofastidiosum*, and *Methanolinea* were the dominant genera in all digestors, with relative abundances accounting for 76.7%-89.2% of the total archaea. Among them, *Methanosaeta* was known as the aceticlastic methanogen (Smith et al., 2007) and accounted for the highest percentage (31.5%) in the control reactors. Its relative abundance increased by 1.32 times with in-sewer Fe(II) dosing and by an even greater 1.53 times with in-sewer Fe(VI) dosing. Similarly, in-sewer Fe(VI) dosing also enriched *Methanobacterium,* a typical hydrogenotrophic methanogen (Zabranska et al., 2018), more significantly than in-sewer Fe(II) dosing (3.05 times v.s. 1.67 times). As for *Candidatus_Methanofastidiosum* (a fastidious methyl-reducing methanogen (Nobu et al., 2016))*,* and *Methanolinea* (a hydrogenotrophic methanogen (Imachi et al., 2008)), the enrichment effect caused by in-sewer Fe(II) and Fe(VI) dosing were similar. In THP-AD, the in-sewer Fe(VI) dosing increased the relative abundances of the four predominant methanogens more significantly by 1.83–3.18 times than the conventional AD. The increase in the relative abundance of methanogens supported the increased methane production with in-sewer iron dosing and THP treatment. But interestingly, the relative abundance of *Candidatus_Methanofastidiosum* was lower in THP-AD, suggesting sludge after THP did not favor its growth. As *Candidatus_Methanofastidiosum* used methylated thiol as the substrate for methane production (Nobu et al., 2016), the decrease in the relative abundance of *Candidatus_Methanofastidiosum* is possibly due to the that the methylated thiol was generated during THP and release from the sludge when the sludge was transferred to the AD (Li et al., 2020). It could also be possible that more readily biodegradable organics were available for methanogenesis after THP (Chen et al., 2018), and the decrease in the relative abundance of *Candidatus_Methanofastidiosum* was a result of the enrichment of other methanogens.

#### 2.3.2 Methanogenic metabolic pathways

**Fig. 4:**
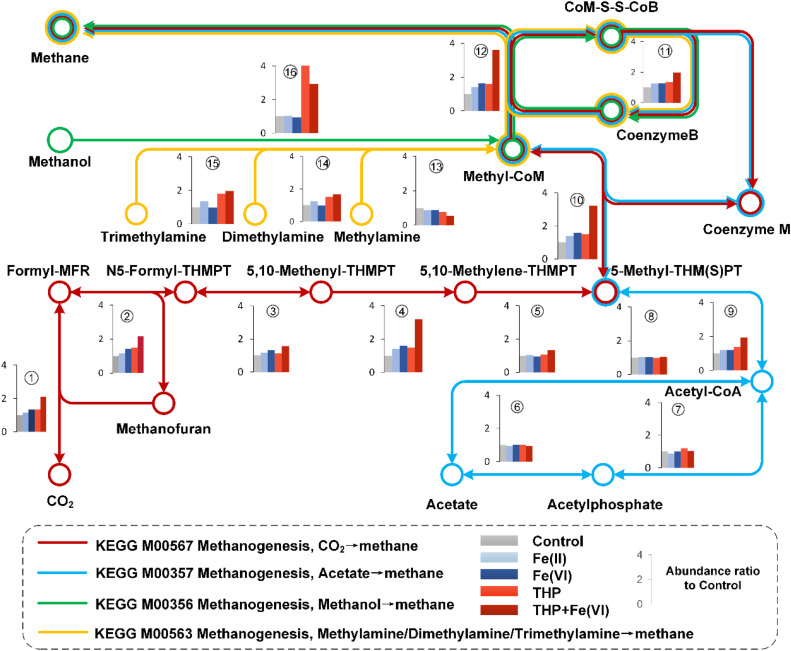
PICRUSt2 predicts microbial pathways and functional characteristics of the methanogenesis process in anaerobic digesters under different reaction conditions. The dots refer to metabolic products and the arrows refer to transformation paths. The inserts compared the total abundances of enzymes detected in each reactor which involved in the corresponding pathway, with the enzyme commission number listed in Y. The abundances of enzymes in the case of Fe(II), Fe(VI), THP, THP-Fe(VI) were presented as the ratios to the control, which were assumed as 1.

The abundances of functional genes involved in four methanogenic pathways (referred to KEGG module) were predicted by PICRUSt2 and summarized in Fig. 4. As shown in Fig. 4, the methanogenesis from CO_2_ (M00567) was enhanced by in-sewer iron dosing, with the enhancement being more significant in the case of Fe(VI) dosing. Specifically, the abundances of functional genes involved in steps in M00567: CO_2_→Formyl-MFR→N5-Formyl-THMPT→5,10-Methenyl-THMPT (Fig. 4, Process 1–4) increased by 15.5%-41.7% with in-sewer Fe(II) dosing and by 31.9% - 59.3% with in-sewer Fe(VI) dosing as compared to the control group. Additionally, for the following steps of 5-Methyl-THM(S)PT→Methyl-CoM→Methane, the gene abundances increased by 40.5%-43.1% and 59.3%-63.2% for in-sewer Fe(II) and Fe(VI) dosing, respectively. Such increases suggested that the methanogenesis pathway using CO_2_ as the substrate was enhanced. In comparison, the methanogenesis through the acetoclastic pathway was not significantly affected by in-sewer iron dosing. This is supported by the comparable abundances of genes involved in the transformation of acetate to acetyl-CoA (Fig. 4, process 6,7 and 8), key steps for acetoclastic methanogenesis. As for methylotrophic methanogenesis (Fig. 4, Process ⑬, ⑭, ⑮ and ⑯), the changes in abundances of functional genes varied from -12.8% to 35.3% with in-sewer iron dosing. However, since the overall abundance of methylotrophic methanogenesis genes was much lower than genes in the other two pathways (Fig. S1), the variation could not affect the overall enhancement effect for methane production.

In terms of THP-AD, improvement in the methanogenesis from CO_2_ by in-sewer iron dosing was more significant than that achieved in conventional AD. The relative abundances of genes in processes 1–4 (Fig. 4) increased by 40.0%-116.4% with the in-sewer Fe(VI) dosing, which is 1.0–2.0 times higher than those achieved in conventional AD. For acetoclastic methanogenesis, the in-sewer iron dosing was also unable to improve the key gene abundances in THP-AD as observed in conventional AD. The genes for the transformation of Acetate→Acetylphosphat→Acetyl-CoA (Process 6–7, Fig. 4) even decreased by 6.9%-12.1% with in-sewer iron dosing in THP-AD. As for methylotrophic methanogenesis, the gene abundances varied from -28.2% to 10.0% with in-sewer iron dosing, but still, they accounted for low proportions (Fig. S1) and were unlikely to affect the overall methane production.

### 2.4 Characterization the resultant particles of in-sewer Fe(II) and Fe(VI) dosing

**Fig. 5:**
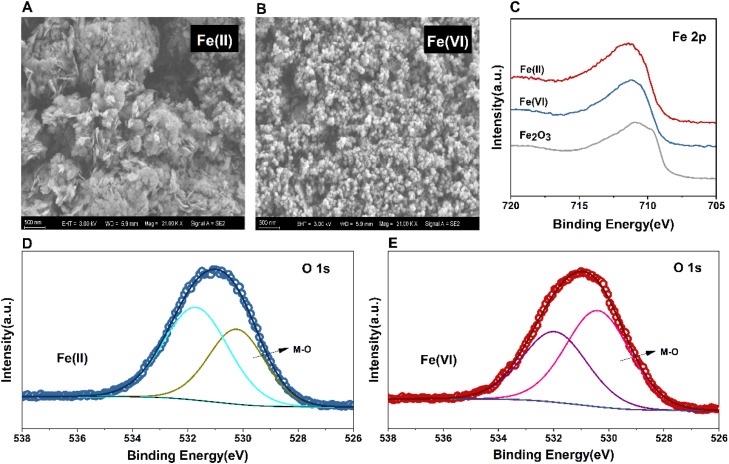
(A) and (B): SEM images of resultant iron particles generated from in-sewer Fe(II) dosing (A) and in-sewer Fe(VI) dosing (B). (C): Fe 2p mapping of XPS of resultant iron particles generated from in-sewer Fe(II) and Fe(VI) dosing and Fe_2_O_3_ standard. (D) and (E): O 1s mapping of XPS of resultant iron particles generated from in-sewer Fe(II) dosing (D) and in-sewer Fe(VI) dosing(E).

The different enhancement effects of in-sewer Fe(II) and Fe(VI) dosing on methane production may be related to the different characteristics of their resultant particles entering the sludge. As shown in scanning electron microscopy (SEM) images (Fig. 5A), Fe(II) resultant particles exhibited an angular sheet-like structure and clustered together densely. In comparison, Fe(VI) resultant particles showed distinctive morphology (Fig. 5B), which were less angular and more rounded. Moreover, the Fe(VI) resultant particles were smaller than the Fe(II) resultant particles and clustered loosely.

The chemical compositions of resultant particles were further determined by X-ray photoelectron spectroscopy (XPS). In the Fe 2p3/2 region, the binding energy peak of the resultant particles generated by in-sewer Fe(II) and Fe(IV) are different at 711.38 eV and 711.18 eV, respectively. The spectra of Fe(VI) resultant particles were closer to Fe_2_O_3_ standards than Fe(II) resultant particles (Fig. 5C). The O1s region spectra of resultant particles were fitted with two separate peaks and compared in Fig. 5, Fig. 5. The binding energy peaks at 528.9–530.0 eV refer to the lattice oxygen, commonly caused by the oxygen of metal oxide (Y. Wang et al., 2019). The binding energy peaks at 533.0 eV often refer to the adsorbed oxygen, which could be resulted from OH^−^ (Chen et al., 2021; Deng et al., 2016). The results showed the peak area of metal oxide in Fe(VI) resultant particles is larger than that of Fe(II) resultant particles. This result further confirmed that more iron oxide was generated by the in-sewer Fe(VI) dosing than Fe(II) dosing.

## 3 Discussion

### 3.1 Mechanistic differences in enhanced methane production by in-sewer Fe(II) and Fe(VI) dosing

**Fig. 6:**
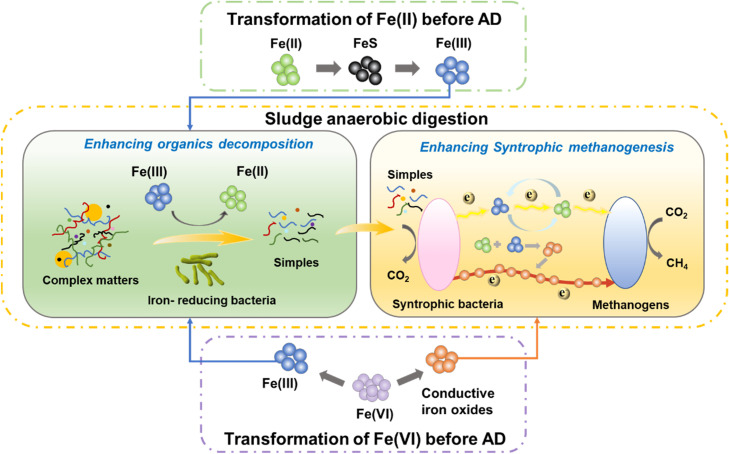
Different mechanisms of in-sewer Fe(II) and Fe(VI) dosing to promote downstream sludge anaerobic digestion processes.

This study showed that both in-sewer Fe(II) and Fe(VI) dosing could promote methane production in downstream sludge AD, with Fe(VI) dosing presenting a higher enhancement effect. This could be related to differences in the transformation route of Fe(II) and Fe(VI) and resultant particle properties after dosed to sewers (Fig. 6).

After Fe(II) was dosed into sewers, it would first react with sulfide forming FeS. When the FeS reached the aeration tank in WWTP, it could be mainly oxidized to Fe(OH)_3_ (Ge et al., 2013). FePO_4_ might also be formed but only accounted for a small fraction, as phosphate concentrations in the wastewater and sludge were much lower than the Fe(II) added in the system. When Fe(OH)_3_ entered the sludge, AD might be improved in two ways: (1) The iron-reducing bacteria were capable of oxidizing more organic compounds under anaerobic conditions using ferric iron as the electron acceptor, thus enhancing the decomposing of complex organics (M. Wang et al., 2019). (2) The direct interspecies electron transfer (DIET) between syntrophic bacteria and methanogen could be enhanced, thus improving methanogenesis. As Fe(OH)_3_ is unconducive, the enhancement of DIET was believed to be triggered by Fe(III) reduction. Tang et al. (2016) found that after the Fe(III) was reduced to Fe(II), the secondary mineralization of Fe(II) with the remained Fe(III) could form conductive magnetite (Fe^II^Fe^III^_2_O_4_) and consequently enhance DIET. Jiang et al. (2013b) provided another explanation as the electron transfer was improved by a Fe(III)(mineral)-Fe(II)-Fe (III)(mineral) redox cycle. Namely, the reduced Fe(II) from Fe(III) could be readsorbed onto the Fe(III) particles, followed by the reprecipitation as structural Fe(III) through simultaneous loss of an electron. Then, the methanogens obtain electrons from Fe(II) for methanogenesis. The improvement of DIET could be supported by the upregulation of the key functional genes in Processes 145 after in-sewer Fe(II) addition, which were recognized genes related to electron transfer between syntrophic bacteria and methanogen (Rotaru et al., 2014). It could also be noted that the relative abundance of *Methanosaeta* was improved by 1.32 times after Fe(II) dosing, but its well-known aceticlastic methanogenesis pathway was not enhanced. The result further confirmed that the electron transfer between syntrophic bacteria and methanogen was enhanced as *Methanosaeta* is one of the model methanogens that could perform DIET (Rotaru et al., 2014).

As for in-sewer Fe(VI) dosing, Fe(VI) would inactivate most SRBs so that limited FeS would be generated (Yan et al., 2020). As a result, Fe(VI) will be mainly reduced to Fe(III) in wastewater. Although it is believed that the Fe(VI) resultant particles contained Fe(OH)_3_, the XPS results in this study suggested it also contains Fe_2_O_3_. The generation of Fe_2_O_3_ from Fe(VI) has also been reported by Goodwill et al. (2015), but the matrix they used was DI water, which is much simpler than that used in this study. As Fe_2_O_3_ is a semi-conductive material, it could be directly used as the electron transfer mediator between syntrophic bacteria and methanogen in AD systems (Kato et al., 2012; Xu et al., 2024). In other words, DIET could be directly enhanced by in-sewer Fe(VI) resultant particles, and it is unnecessary to wait for the driving of microbial Fe(III) reduction as in the case of in-sewer Fe(II) dosing. In addition, probably due to the different reaction routes, the Fe(VI) resultant particle had a smaller sized and looser structure than Fe(II) resultant particle, which made it more easily to contact and interact with microorganisms. This may also contribute to the more effective enhancement of methane production caused by in-sewer Fe(VI) dosing (Goodwill et al., 2015).

### 3.2 In-sewer iron dosing as a promising strategy facilitating the energy- and carbon neutralization in urban water management

The results of this study suggested that in-sewer iron dosing could not only solve the sewer odour and corrosion problems (Ganigue et al., 2011) but also enhance the bioenergy (methane) recovery from the sewage sludge under different conditions. Nowadays, with emphasis on the energy- and carbon neutralization in urban water management, pretreatment methods, such as THP, have been increasingly applied to improve the bioenergy recovery from sludge AD (Mills et al., 2014). Our results showed that the enhancement of cumulative methane production by in-sewer iron dosing achieved in THP-AD is higher than that in conventional AD. This result suggested that the in-sewer iron dosing was in line with the current development trend of sewage sludge AD (Ampese et al., 2022). The higher improvement in THP-AD could be explained by that the THP improved the disintegration and hydrolysis of the complex organics in sewage sludge (Barber, 2016), so that more organics were readily for syntrophic bacteria and methanogen for methanogenesis which was reinforced by in-sewer iron dosing. With the improved total methane production as well as the methane production rate achieved by in-sewer iron dosing, the sludge retention time (SRT) in an anaerobic digestor would be reduced.

The result of this study further indicated that the in-sewer Fe(VI) dosing was more effective for enhancing bioenergy recovery from sewage sludge than Fe(II) by the possible mechanisms discussed in Section 3.1. In fact, there were additional benefits of in-sewer Fe(VI) dosing for energy- and carbon-neutral urban water management. Our previous study suggested that in-sewer Fe(VI) could effectively reduce the methane emissions in sewers and prevent sewer pipe corrosion, as it could inactivate the microbial activities in sewers (Yan et al., 2020). Therefore, it could be expected that more organics in wastewater could be preserved during transportation and reach the downstream WWTP for bioenergy recovery. Thus, in-sewer Fe(VI) dosing could be regarded as a promising integrated wastewater management strategy facilitating the energy- and carbon-neutralization of urban water systems. As for practical applications, future efforts should be dedicated to improving Fe(VI) manufacturing with a higher purity and low carbon footprint. Also, the performance of a semi-continuous anaerobic digestor should be studied and VS reduction should be tested to provide more understanding of the impact of in-sewer Fe(VI) dosing for long-term operation. Additionally, studies on optimizing Fe(VI) dosing strategies from the perspective of integrated management of sewer networks and WWTP along with whole life-cycle carbon footprint assessment, are essential for fully evaluating and enhancing energy and carbon neutralization effects.

## 4 Conclusions

This study investigates the impact of in-sewer Fe(II) and Fe(VI) dosing on sludge AD in downstream WWTP and the impact of THP was also studied for the in-sewer Fe(VI) dosing case. The following conclusion could be drawn.

(1) Both in-sewer Fe(II) and Fe(VI) dosing could promote methane production in downstream sludge AD, with Fe(VI) dosing presenting a higher enhancement effect.
(2) The promotion effect is primarily attributed to enhanced direct interspecies electron transfer between syntrophic bacteria and methanogen, with the smaller size and higher iron oxide content in Fe(VI) resultant particles being more conducive to such processes.
(3) The enhancement in methane production resulting from in-sewer iron dosing in THP-AD is higher than that achieved in conventional AD, indicating that in-sewer iron dosing supports the growing application of THP-AD for improved bioenergy recovery from sludge.
(4) In-sewer Fe(VI) dosing appears more promising within integrated wastewater management strategies for facilitating the energy-neutralization of urban water systems, due to higher methane production in AD.

## 5 Materials and methods

### 5.1 Wastewater and sludge

Domestic wastewater used in this study was collected every three days from a wet well in Yangpu District, Shanghai, China. The wastewater typically contained sulfide concentrations of 1–2 mg S/L, sulfate concentrations of 30–40 mg S/L, VFA concentrations of 50 -100 mg COD/L, SCOD concentration of 100–170 mg/L, TCOD concentration of 300∼450 mg/L, ammonia concentration of 30–35 mg/L and phosphate concentration of 2–4 mg/L. Once collected, it was immediately transported to the laboratory and stored at 4 °C under refrigeration.

The activated sludge to simulating the wastewater treatment processes and feeding the anaerobic digestor taken from a secondary sedimentation tank in Shanghai Bailonggang Wastewater Treatment Plant. The sludge has a total solids (TS) of 8.9 ± 0.1 g/L and volatile solids (VS) of 3.1 ± 0.1 g/L, pH of 6.8 ± 0.2, TCOD of 5.8 ± 0.2 g/L, SCOD of 610 ± 35 mg/L, ammonia of 2.4 ± 0.2 mg/L and phosphate of 5.9 ± 0.5 mg/L and was stored at 4 °C after collection before the experiments were carried out in 2–3 days. The inoculum for the AD experiments was gained from a laboratory scale semi-continuous AD reactor operated at 35 °C (TS: 30.9 ± 0.1 g/L and VS: 16.1 ± 0.1 g/L).

### 5.2 Simulating the reaction processes of different iron salts in sewers and wastewater treatment plants

Once the iron salt was dosed in sewers, it would be transformed in the sewer system and then in activated sludge processes before reaching the anaerobic digestor. Therefore, the transformation of different types of iron salts in the sewer and activated sludge process were simulated first. Then the iron-containing sludge was transferred for AD with or without THP. Overall, five cases combining different iron dosing and sludge AD conditions were studied (Table 2), with detailed experimental designed for each transformation process and sludge AD described below.

**Table 2:** Experimental cases for different in-sewer iron dosing and sludge anaerobic digestion conditions.

Case	K_2_FeO_4_ (mgFe)	FeCl_2_ (mgFe)	Sulfide (mgS)	Anaerobic digestion conditions	Lable
1	0	0	0	Conventional AD	Control
2	0	100	57.5	Conventional AD	Fe(II)
3	100	0	10	Conventional AD	Fe(VI)
4	0	0	0	THP-AD	THP
5	100	0	10	THP-AD	THP-Fe(VI)

#### 5.2.1 Transformation of iron salts in the sewer

The experiments to mimic the transformation of Fe(II) and Fe(VI) in the sewer system were conducted in five 500 mL reactors (referred to as F_1_-F_5_) filled with wastewater. K_2_FeO_4_, FeCl_2_, and Na_2_S were then added to each reactor according to the mass shown in Table 2. It is assumed that the hydraulic retention time (HRT) of wastewater in WWTP was typically 0.5 days, and the SRT was 10 days (Kargi et al., 2007; Sun et al., 2021). The masses listed in Table 2 were determined based on the reported dosing rate that can effectively control sulfide in sewers and the total Fe and S that can be accumulated in the sludge during an SRT (10 days) (Ge et al., 2013). Specifically, 100 mg Fe K_2_FeO_4_ used was equivalent to a typical dosage of 100 mg Fe/L K_2_FeO_4_ in sewers and the dosing interval was five days (Yan et al., 2020). The slow rate of sulfide generation after Fe(VI) addition resulted in a cumulative sulfide content of 10 mg over 10 days, assuming that the sulfide concentration in the wastewater was controlled at 1 mgS/L and the HRT of 0.5 days. As for the in-sewer Fe(II) dosing case, a content of 100 mg Fe(II) used in this experiment was equivalent to a typical daily dose of 10 mg Fe(II)/L in the sewer system (Gutierrez et al., 2010; Liu et al., 2023). Since Fe(II) only precipitates hydrogen sulfide and would not alter the rate of sulfide production by the sewer biofilm, the cumulative sulfide over 10 days according to the stoichiometry of the chemical reaction (Fe^2+^+S^2−^=FeS) (Ganigué et al., 2018) can reach 57.4 mgS. It should be noted that the commercialized K_2_FeO_4_ (Analytical Reagent) was used in this study but the chemical was tested containing Fe(III) of 42%. The low purity is a common problem of most commercialized K_2_FeO_4_ reagent (Li et al., 2005). Therefore, to ensure the same total Fe being added to each reactor, the Fe added in K_2_FeO_4_ cases (Case 3 and Case 5) were determined based on total Fe in the reagent. As the Fe(III) was mainly the product of Fe(VI) reduction. Its potential impact on AD should follow similar mechanisms as the in-sewer Fe(VI) dosing. This is because when Fe(VI) was dosed into sewers, it would be reduced first before entering the anaerobic digestor. After all the chemicals were added, all reactors were fully cover to avoid the loss of H_2_S due to emission or oxidation by air. The mixtures were gently mixed using a magnetic stirrer for 30 min. In this case, the water with Fe(II) dosing turned black, indicating the formation of FeS precipitates.

#### 5.2.2 Transformation of iron salts in activated sludge process

The products generated by in-sewer iron dosing would flow into the WWTP. Some FeS precipitates could be re-oxidized in the aeration tank in activated sludge processes. This process was simulated in five 1L reactors (referred to as O_1_-O_5_), receiving the products generated in F_1_-F_5_, respectively. For the experimental setup, each reactor was firstly filled with 500 mL of activated sludge as described in Section 5.1 and flushed with nitrogen gas (99.99%) for 0.5h to remove any oxygen present. Subsequently, the sludge was allowed to settle by leaving it under static conditions for 1 hour, and 200 ml of supernatant was discarded from each reactor. Then, 300 mL of well-mixed solution from each of the previously prepared F_1_-F_5_ was added to O_1_-O_5_. As a result, the VS in the system was ∼ 2.6 g/L, which was in the range of the VS commonly used for activated sludge processes (Tchobanoglous et al., 2014). The sludge mixture was allowed to continue aeration for 12 h at a rate of 0.5 L/min until the FeS precipitates were fully oxidized, based on the FeS re-oxidation rates shown in the study by Gutierrez et al. (2010). Finally, the mixture in O_1_-O_5_ was allowed to settle for 1 h and 100 mL of the supernatant was discarded to retain the thickened waste activated sludge (WAS). The remaining sludge was then used in the following AD experiments.

#### 5.2.3 Anaerobic digestion experiments

The AD of WAS obtained in O_1_-O_5_ were carried out in 120 mL serum bottles and referred to as R_1_-R_5_, respectively. R_1_-R_3_ were carried in conventional AD and are hereinafter named Control, Fe(II), and Fe(VI), respectively. For R_4_-R_5_ in THP-AD were applied and are hereinafter named THP, THP+Fe(VI), respectively. For THP experiments, WAS obtained from O_4_ and O_5_ was treated at 140 °C separately in a 1.5 L stainless-steel pressure vessel with electrical heating (EasyChem E500, Beijing, China) for 30 min (Tang et al., 2022). After 30 min, the cooling procedure started. When the temperature was reduced to below 60 °C, the reactors were opened, and the slurry was cooled down in ice water (taking 20−30 min). Before AD, the VFA, SCOD, TCOD and the VS concentrations were measured in the feeding sludge in each reactor. For the AD setup, each glass vial was filled with 80 mL of sludge to form a headspace of 40 mL. The 80 mL of sludge consisted of approxately 60 mL of WAS obtained from O_1_-O_3_ and THP treated WAS from O_4_-O_5_ depinding on the VS concentration, as well as 20 mL of digested sludge for digested sludge, resulting in the ratio of VS of activated sludge to digested sludge in each reactor 2:1. Distilled water was added to adjust the total volume of the sludge being the same in each vial. After loading the sludge, the bottles were sealed with butyl rubber stopper with aluminum cover after the headspace flushed by nitrogen gas (99.99%) to deoxygenation. Then, all the bottles were placed into a 35 °C shaking incubator with a mixing speed of 200 rpm.

Biogas samples were taken every 1–3 days to measure methane, CO_2,_ and H_2_ concentrations. Before sampling, the bottles were inverted and shaken to mix the sludge well so that the gas produced by the sludge could fully enter the gas phase. The methane production was reported as the volume of methane produced per gram of VS added (mLCH_4_∙gVSadded^−1^). In addition, serum bottles with the same sludge composition and operational conditions as described above were set up for liquid sample analysis every five days. During each sampling time, 1 ml of liquid was withdrawn and diluted for VFA determination. For each group, the experiments were conducted in triplicate.

### 5.3 High-throughput sequencing

In order to investigate the effect of in-sewer iron salts dosing on the microbial diversity in the downstream AD reactor, the digested sludge in the AD reactor R_1_-R_5_ was taken at the end of the experiment for high-throughput sequencing analysis. The specific operations were as follows. Firstly, the DNA samples of digested sludge were extracted by a E.Z.N.A.® soil DNA kit (Omega Bio-tek, Norcross, GA, U.S.) and then detected by agarose gel electrophoresis and Nano Drop 2000. Secondly, PCR amplification of the V3-V4 region of the 16S rRNA gene using 515FmodF (5′-GTGYCAGCMGCCGCGGTAA-3′) and 806RmodR (5′-GGACTACNVGGGTWTCTAAT-3′). The PCR products were extracted using the AxyPrep DNAGel Extraction Kit (Axygen Biosciences, Union City, CA, USA), detected by 2% agarose gel electrophoresis and quantified by Quantus™ Fluorometer (Promega, USA). Databases were constructed using the NEXTFLEX Rapid DNA-Seq Kit and the PCR products were subjected to Illumina Miseq PE300 sequencing. Finally, the optimized sequences after QC splicing are processed for noise reduction using the DADA2 (Sturm et al., 2014) plugin in the Qiime2 process. Sequences after DADA2 noise-reducing are often referred to as ASVs (amplicon sequence variants). Chloroplast and mitochondrial sequences annotated to all samples were removed, and then the number of sequences in all samples was flattened to 20,000. After flattening, the average sequence coverage (Good's coverage) for each sample was still up to 99.09%. ASVs were analyzed for species taxonomy based on the Sliva 16S rRNA gene database (v 138) using the Naive Bayes classifier in QIIME2. Diversity data and the community structure at each classification level according to the results of microbial ASVs cluster analysis and species taxonomy analysis. The raw reads of all tested samples were deposited into the NCBI Sequence Read Archive (SRA) database (Accession Number: PRJNA1164677). PICRUSt2 analysis was adopted for predicting metagenome information using the 16S rRNA sequencing data (Douglas et al., 2020). Predictions were made by corresponding the marker gene data and the reference genomes in databases, Kyoto Encyclopedia of Genes and Genomes (KEGG, https://www.kegg.jp/).

### 5.4 Characterizing the resultant iron particles of in-sewer Fe(II) and Fe(VI) dosing

The resultant iron particles of in-sewer Fe(VI) and Fe(II) dosing were characterized using SEM and XPS. The particles were obtained by repeating the experiments simulating the iron transformation in sewers and activated sludge processes as described in 5.2.1 and 5.2.2. But, to avoid the interference of sludge during the characterizations, the activated sludge was not applied in this experiment. The resultant iron particles were then collected through centrifugation and frozen in a refrigerator at -80 °C for 48 hours. Subsequently, the frozen samples were cold-dried in a freeze dryer and finally ground to homogeneity using an agate mortar and pestle before SEM and XPS analysis.

SEM micrographs were taken using a Zeiss Sigma 300 SEM, operating at an accelerating voltage of 3 kV, and a working distance ranging from 4 to 6 mm. Due to the strong magnetic properties of this sample, gold sprayed of the powder was applied before it being mounted on the SEM sample stage. Ten images of each sample were taken and visually assessed for significant differences in morphology. XPS analysis was performed using a Thermofisher Nexsa spectrometer with a monochromatic Al Kα X-ray operating at 1486.6 eV, 37.5 W. The diameter of the analysis area was approximately 400 μM and the step size was 0.1 eV XPS was also performed on a Fe_2_O_3_ standard (hematite, ≥99% purity).

### 5.5 Chemical and statistical analysis

The parameters such as TS, VS, TCOD and SCOD were analyzed according to standard methods (APHA, 2012). The compositions of biogas (CH_4_, CO_2_ and H_2_) was analyzed by the gas chromatograph (GC-SP6890, Shandong Lunan Ruihong Chemical Instrument Co. Ltd., China) equipped with a thermal conductivity detector (TCD). VFA concentration was measured using gas chromatography (Shimadzu GC, 2010 plus) equipped with a flame ionization detector (FID) equipped with a 30 m × 0.32 mm × 0.1 μm Stabilwax-DA polyethylene glycol capillary column (Wu et al., 2021b). One-way analysis of variance (ANOVA) was used for statistical analysis and the P value <0.05 was considered to be significantly different.

The R_max_ and G_0_, two key parameters associated with methane production from WAS, were used to evaluate and compare methane production kinetics and potential of the WAS for the five groups in the above experiment. They were estimated by fitting the methane production data from biochemical methane potential (BMP) tests to a Gompertz model using a modified version (Eq.(1)) (Kafle et al., 2016), with the assumption of methane production rate in a batch digester corresponding to the specific growth rate of methanogen.

(1) $$G\left(t\right)=G_{0}\cdot exp\left\{-exp\left[\frac{R_{max}\cdot e}{G_{0}}\left(\lambda -t\right)+1\right]\right\}$$

Where,

G_0_ = methane production potential (mL/g VS),

R_max_ = maximum methane production rate (mL/(g VS·d)),

λ = lag phase (day), representing the minimum time taken to produce biogas or taken for bacteria to acclimatize to the environment, t = time (day), e = exp (1) = 2.7183.

## CRediT authorship contribution statement

**Jingya Xu:** Writing – original draft, Methodology, Investigation, Formal analysis. **Yizhen Wang:** Methodology, Investigation. **Yanzhao Wang:** Investigation, Formal analysis. **Lai Peng:** Writing – review & editing, Funding acquisition, Conceptualization. **Yifeng Xu:** Writing – review & editing, Conceptualization. **Hailong Yin:** Writing – review & editing. **Bin Dong:** Writing – review & editing. **Xiaohu Dai:** Writing – review & editing, Supervision. **Jing Sun:** Writing – review & editing, Supervision, Project administration, Funding acquisition, Conceptualization.

## Declaration of competing interest

The authors declare that they have no known competing financial interests or personal relationships that could have appeared to influence the work reported in this paper.

## Data Availability

Data will be made available on request

## Appendix Supplementary materials

Image, application 1
