# Supplementary material for: In-sewer iron dosing enhances bioenergy recovery in downstream sewage sludge anaerobic digestion: The impact of iron salt types and thermal hydrolysis pretreatment
Source: Water Res X. 2024 Oct 29;25:100273. doi: 10.1016/j.wroa.2024.100273 (PMC11570779; doi:10.1016/j.wroa.2024.100273)
Supplement: Supplementary file 1 [file mmc1.docx]

**Supporting Information**

**In-sewer iron dosing enhances bioenergy recovery in downstream sewage sludge anaerobic digestion: the impact of iron salt types and thermal hydrolysis pretreatment**

Jingya Xu^a^, Yizhen Wang^a^, Yanzhao Wang^a^, Lai Peng^b,d^, Yifeng Xu^b,d^, Hailong Yin^a,c^, Bin Dong^a,c^, Xiaohu Dai^a,c^, Jing Sun^a,b,c*^

a. State Key Laboratory of Pollution Control and Resource Reuse, School of Environmental Science and Engineering, Tongji University, Shanghai, 200092, China

b. Key Laboratory of Yangtze River Water Environment, School of Environmental Science and Engineering, Tongji University, Shanghai, 200092, China

c. Shanghai Institute of Pollution Control and Ecological Security, Shanghai, 200092, China

d. Key Laboratory of Green Utilization of Critical Non-metallic Mineral Resources, Ministry of Education, Wuhan University of Technology, Wuhan 430070, China

**Table S1.** Enzymes involved in methanogenesis pathways ①- ⑯ as presented in Figure. 4 according to KEGG database.

| No. | Pathway description | Enzymes involved |
| --- | --- | --- |
| 1 | CO2 → Formyl-MFR | 1.2.7.12 |
| 2 | Formyl-MFR → N5-Formyl-THMPT | 2.3.1.101 |
| 3 | N5-Formyl-THMPT ↔5,10-MethenylTHMPT | 3.5.4.27 |
| 4 | 5,10-Methenyl-THMPT ↔ 5,10- Methylene-THMPT | 1.12.98.2;1.5.98.1 |
| 5 | 5,10-Methylene-THMPT → 5-MethylTHM(S)PT | 1.5.98.2 |
| 6 | Acetate ↔Acetylphosphate | 2.7.2.1 |
| 7 | Acetylphosphate ↔Acetyl-CoA | 2.3.1.8 |
| 8 | Acetate ↔Acetyl-CoA | 6.2.1.1 |
| 9 | Acetyl-CoA ↔5-Methyl-THM(S)PT | ACDS |
| 10 | Methyl-CoM ↔5,10-Methyl-THM(S)PT + Coenzyme M | 7.2.1.4 |
| 11 | CoM-S-S-CoB → Coenzyme B | 1.8.7.3;1.8.98.1;1.8.98.5;1.8.98.6;1.8.7.3;1.8.98.4 ;1.8.98.5;1.8.98.6;1.8.98.1 |
| 12 | Coenzyme B + Methyl-CoM → CoM-S-S-CoB + Methane | 2.8.4.1 |
| 13 | Methylamine → Methyl-CoM | MfbA MtmB MtmC |
| 14 | Dimethylamine → Methyl-CoM | MfbA MfbB MfbC |
| 15 | Trimethylamine → Methyl-CoM | MfbA MttB MttC |
| 16 | Methanol → Methyl-CoM | MtaA MtaB MtaC |

**
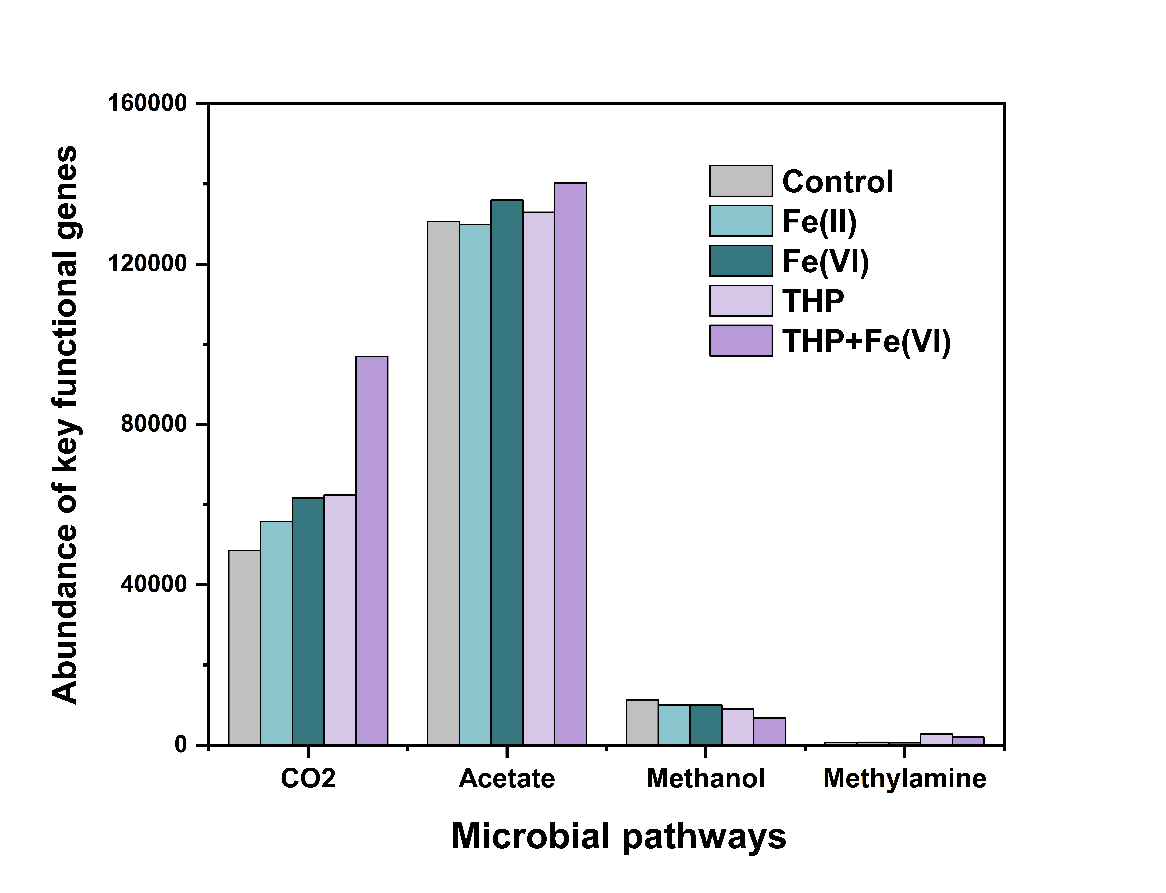
**

**Figure S1.** Overall abundance of key functional genes in four methane-producing pathways in different reactors.CO_2_ refers to the production of methane through the CO_2_ conversion pathway(①-⑤).Acetate refers to the production of methane through the acetate conversion pathway(⑥-⑨).Methanol refers to the production of methane through the acetate conversion pathway(⑯). methylamine refers to the production of methane through the methylamine/dimethylamine/trimethylamine conversion pathway(⑬-⑮).
